# Endothelial dysfunction in adipose triglyceride lipase deficiency: role of perivascular adipose tissue

**DOI:** 10.1186/2050-6511-14-S1-P45

**Published:** 2013-08-29

**Authors:** Marion Mussbacher, Karoline Pail, Gerald Wölkart, Günter Hämmerle, Alois Lametschwandtner, Rudolf Zechner, Bernd Mayer, Astrid Schrammel

**Affiliations:** 1 Department of Pharmacology and Toxicology, Institute of Pharmaceutical Sciences, University of Graz, 8010 Graz, Austria; 2 Department of Molecular Bioscience, University of Graz, 8010 Graz, Austria; 3 Department of Cell Biology and Physiology, Vessel and Muscle Research Unit, University of Salzburg, 5020 Salzburg, Austria

## Background

Perivascular adipose tissue (PVAT) has been shown to be an important modulator of vascular function through release of both relaxing and contracting factors. However, the involvement of PVAT in development of endothelial dysfunction is not well understood, yet. We have recently demonstrated that mice lacking adipose triglyceride lipase (ATGL) suffer from severe endothelial dysfunction. Since vessels of these mice are coated with large amounts of PVAT, we speculated that this might potentially contribute to disturbed vascular homeostasis. Therefore, PVAT of wild type (WT) and ATGL knockout mice was characteri-zed in terms of inflammatory as well as oxidative stress. Additionally, we wanted to distinguish between short-term PVAT-mediated effects on vascular function as well as long-term *in vivo* interference of PVAT with aortic NO/cGMP signaling.

## Results

Knockout of ATGL resulted in 7-fold increase in PVAT wet weight. Adipose mRNA levels of the inflammation markers tumor necrosis factor α (TNFα, monocyte chemoattractant protein 1 (MCP-1) and interleukin 6 (IL-6) were significantly increased in ATGL-deficient PVAT. In addition, NADPH oxidase isoform NOX2 as well as its cytosolic subunit p67phox were significantly upregulated at both mRNA and protein level, indicating the presence of oxidative stress. Massive infiltration of macrophages was confirmed by a tremendous increase of galectin-3 (Mac-2) protein expression. Performing isometric tension vasomotor studies, we found that PVAT did not affect aortic relaxation to the endothelium-dependent agonist acetylcholine or to the NO donor 2,2-diethyl-1-nitroso-oxyhydrazine (DEA/NO) in WT and ATGL knockout mice. By contrast, presence of PVAT reduced the contractile efficiency of the thromboxane mimetic 9,11-dideoxy-11α, 9α-epoxy-methanoprostaglandin Fα (U46619) in WT aortas (from 155.1±5.7% to 125.6±9.4%). This effect was even more pronounced in ATGL-deficient aortas (162.9±5.5% to 90.4±9.6%), suggesting presence of a PVAT-derived relaxing factor.

**Figure 1:**
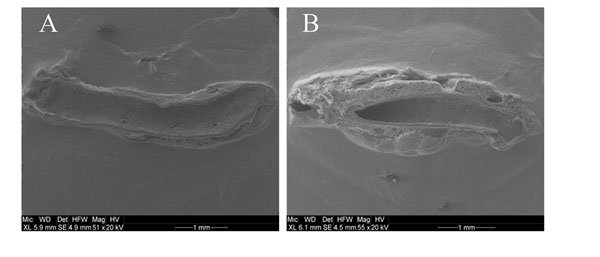
Thoracic aortas of WT (A) and ATGL(-/-) (B) mice were fixated by perfusion with glutaraldehyde *in situ* and analyzed by electron scanning microscopy.

## Conclusion

These data suggest that PVAT-derived inflammatory and oxidative stress might contribute to endothelial dysfunction in ATGL deficiency. Further studies are necessary to identify the PVAT-derived relaxing factor and its role in endothelial (dys)function. To address *in vivo* functional consequences of PVAT on vascular function, a mouse model of adipose tissue-specific rescue of ATGL is currently created.

